# Meanings attributed to changes experienced by individuals after COVID-19 hospitalization

**DOI:** 10.1590/0034-7167-2023-0122

**Published:** 2024-08-30

**Authors:** Camila Harmuch, Jéssica dos Santos Pini, Paula Antunes Bezerra Nacamura, Anny Caroline Ribeiro Devechi, Vinícius Brito de Souza, Juliana Graciela Vestena Zillmer, Maria Aparecida Salci, Marcelle Paiano

**Affiliations:** I Universidade Estadual de Maringá. Maringá, Paraná, Brazil; II Universidade Federal de Pelotas. Pelotas, Rio Grande do Sul, Brazil

**Keywords:** COVID-19, Life Change Events, Hospitalization, Grounded Theory, Nursing, COVID-19, Acontecimientos que Cambian la Vida, Hospitalización, Teoría Fundamentada, Enfermería

## Abstract

**Objectives:**

to understand the meanings attributed to the experiences of individuals after hospitalization for COVID-19.

**Methods:**

qualitative study, with a theoretical framework based on Symbolic Interactionism and a methodological approach grounded in Grounded Theory. Nineteen participants who had moderate and severe forms of COVID-19 after hospitalization were interviewed. Data collection took place between April and November 2021 through online interviews, and the data were analyzed using initial and focused coding in the MAXQDA software.

**Results:**

the data illustrate new meanings attributed to different aspects of life after hospitalization, including relationships with others, the environment, physical and mental health, finances, identity, and interactions with a new social reality.

**Conclusions:**

the meanings are intrinsically linked to the value of interpersonal relationships, the perception of their impact, and the consequences after hospitalization. This allows professionals to understand the importance of this information to improve care and prepare for future epidemics.

## RESUMEN

**Objetivos:**

comprender los significados atribuídos a las experiencias vividas por indivíduos después de la hospitalización por COVID-19.

**Métodos:**

estudio cualitativo, con referencia teórica en el Interaccionismo Simbólico y metodológica en la Teoría Fundamentada en los Datos. Se entrevistaron a 19 participantes con formas moderadas y graves de COVID-19 después de la hospitalización. La recolección de datos tuvo lugar entre abril y noviembre de 2021, mediante entrevistas en línea, y los datos se analizaron siguiendo la codificación inicial y focalizada, utilizando el software MAXQDA.

**Resultados:**

los datos ilustran nuevos significados en diferentes aspectos de la vida después de la hospitalización, tales como en la relación con otros, con el entorno, la salud física y mental, finanzas, identidad e interacciones con una nueva realidad social.

**Consideraciones Finales:**

los significados están intrínsecamente ligados a la valorización de las relaciones interpersonales, la percepción de su impacto y las consecuencias después de la hospitalización, permitiendo a los profesionales reconocer la importancia de esta información para mejorar el cuidado y prepararse para enfrentar futuras epidemias.

## INTRODUCTION

Individual experiences during hospitalization for COVID-19 were impacted by various factors, such as the presence of symptoms, the duration and stage of hospitalization, the severity of the disease, social changes, access to information, and previous experiences^(1-2)^.

Prevailing emotions included fear, denial, stigma, and a sense of abandonment, especially at the onset of symptoms, upon hospital admission, and during isolation. These were accompanied by uncertainty, worry, and expectations regarding prognosis after diagnosis confirmation. Acceptance of the clinical situation and treatment occurred over the course of hospitalization and near discharge^(2)^.

During hospitalization, bodily reactions were also influenced by the lived experience, reflecting in excessive attention to symptoms, changes in diet, sleep, and behavior. In some cases, the illness resulted in psychological growth and changes in addressing daily challenges due to the newfound appreciation of life, family, courage, and tenacity^(3)^.

Regarding the severity of COVID-19, which is associated with clinical symptoms, a qualitative study with 24 patients revealed that during hospitalization, those with mild to moderate disease faced uncertainties regarding progression, clinical management, and recovery, while more severe cases dealt with these issues along with the fear of death^(2)^. Additionally, participants hospitalized in the Intensive Care Unit (ICU) expressed a need to understand what occurred during this period, as fear, anguish, and pain persisted for months after discharge^(4)^.

People who were hospitalized due to COVID-19 became extremely vulnerable, with new health needs after this period^(5-6)^, such as post-traumatic stress syndrome, depression, and reduced quality of life^(7-8)^. These new demands make managing this disease more challenging for both the patient and their families, as well as for health services^(9)^.

Therefore, it is considered that experiences are interpreted according to the symbols that arise in a specific situation, unique to each person and their social interactions. Thus, the theory of Symbolic Interactionism (SI) helps explain the dynamic processes that occur in the relationships between the individual and the elements of interaction after hospitalization^(10)^.

To collect and analyze information with the aim of deeply understanding the complex personal meanings and experiences that arise with hospitalization for COVID-19, Grounded Theory (GT) is used. Through it, insights applicable to health care, interventions, and policies can be obtained, making them more effective and patient-centered. This method allows for a holistic research approach, which is vital for fully understanding the impact of COVID-19 hospitalization on people’s lives and how the health team can best support them.

From this perspective, by identifying the experiences of people hospitalized for COVID-19 and their meanings, it is possible to establish more effective management of this disease and improve health care during and after hospitalization. This, in turn, helps facilitate an effective and timely return of these individuals to their pre-pandemic life routines.

## OBJECTIVES

To understand the meanings attributed to the experiences of individuals after hospitalization for COVID-19.

## METHODS

### Ethical Aspects

The development of the study complied with the ethical principles set forth in Resolution 466/12 of the National Health Council. It was approved by the Permanent Committee on Ethics in Research Involving Human Beings as part of a cohort study. All interviews were conducted with informed consent, and the Free and Informed Consent Form was signed.

The interviewees were identified by their ward, followed by Arabic numerals, according to the order of the interviews (“ward 1,” “ward 2,“ ”ICU 1,” “ICU 2”), ensuring confidentiality and anonymity throughout the research process. The choice of ward as an identifier allows for the assessment of the severity of the participant’s condition, as more severe patients were hospitalized in the ICU.

### Theoretical-Methodological Framework

SI was used as a theoretical perspective to support the development of the research and help understand the different social and intrapersonal interactive processes of individuals hospitalized for COVID-19. Consequently, the use of GT^(11)^ is justified as it enables an in-depth understanding of underexplored areas and allows for a detailed analysis of the phenomenon under study.

### Study Type

This is a qualitative study conducted based on the guidelines proposed by the Consolidated Criteria for Reporting Qualitative Research (COREQ)^(12)^.

### Study Setting

The study was conducted in a medium-sized municipality located in the northwest region of Paraná, with a population of 409,657 people and a territorial area of 487.012 km^2^, according to the 2022 Census^(13)^. The number of confirmed COVID-19 cases in the region, as of December 31, 2020, was 34,797 cases, with 26,502 recoveries and 537 deaths. The number of hospitalizations of adults and elderly individuals in 2020 was 1,830^(14)^.

### Participants and Data Source

The participants were adults residing in the study municipality who were discharged from the hospital after being admitted to the ICU or wards. The sample was selected following the concept of theoretical sampling proposed by GT. The theoretical sampling process occurred through constant comparative analysis of the data, guiding the researcher to collect specific information to fill conceptual gaps. Theoretical sampling is not predetermined; it is defined during data analysis based on the needs evidenced by the theory and the theoretical saturation of the data. In other words, as information is repeated and no new data are found in a particular group, the search is halted^(11)^. At the end of this process, the sample consisted of 19 individuals.

For the formation of the sampling groups, criteria were established based on the theoretical sampling process proposed by GT. Three sampling groups were constituted. For the selection of the first sampling group, the following inclusion criteria were adopted: adults who had been hospitalized and discharged from wards between March 2020 and June 2020, with a final classification of moderate or severe COVID-19, and who had valid phone numbers. Those who did not answer phone calls after three consecutive attempts were excluded.

The first sampling group consisted of seven participants and aimed to understand their experiences during the hospitalization process. As data collection and analysis progressed, questions arose about the influence of the months in which hospitalization occurred. This is due to the fact that, throughout 2020, there were fluctuations between stability and peaks of the disease, which could be related to the experiences presented.

Thus, it was decided to continue data collection with participants who had been hospitalized in wards but in different months from the first sampling group, in order to understand the phenomenon under study in greater depth. Therefore, the second sampling group consisted of six individuals hospitalized in wards between September and December 2020, following the same inclusion criteria as the first sampling group.

With the continuation of new experiences from the interviewees, it was decided to form the third sampling group, and the following hypothesis was raised: besides the hospitalization period, does the hospitalization sector have the potential to influence the meanings attributed to illness, hospitalization, and discharge? Thus, for the third sampling group, the following inclusion criterion was established: individuals classified as moderate or severe COVID-19, with valid phone numbers, who had been hospitalized in the ICU at any time in 2020 and had been discharged from the hospital.

A total of 46 potential participants in the three sampling groups were contacted via phone. Of these, 17 were excluded for not answering after three contact attempts, eight refused to participate in the research due to lack of time and interest, and two were unable to participate due to mental and physical conditions, as reported by themselves or a family representative.

### Data Collection and organization

The list of potential participants was compiled from the database of a prospective cohort study involving individuals who were hospitalized due to COVID-19. This database contained all the information from the mandatory disease notification forms, and the inclusion and exclusion criteria were applied in its preparation. The researcher, a nurse trained in data collection, contacted potential participants by phone, establishing rapport and explaining the study’s reasons and objectives. She then presented the data collection objectives and methods, inviting them to participate.

Upon agreement, an interview was scheduled and conducted individually via video call using the WhatsApp® application by the principal researcher. Participants were asked to be in a private and quiet environment of their choice to facilitate communication and make their participation more comfortable. The reflections and expressions of the interviewees were recorded in a field diary, and the audio of the interviews was recorded with the participants’ consent. It should be noted that the online interviews were conducted in compliance with the recommendations of the Research Ethics Committee, whose biosafety guidelines aimed to minimize the risk of COVID-19 transmission during data collection.

Data collection for the first sampling group occurred between April and June 2021; for the second group, between July and September 2021; and for the third group, between October and November 2021. The following guiding question was used: “What has changed in your life after being discharged from the hospital for COVID-19?”. Additional support questions common to all sampling groups included: What did hospitalization mean to you? What did hospital discharge mean to you? What changes occurred in your life after diagnosis and hospitalization? How did you cope with these changes? How have things been for you after discharge? How are your relationships with others?

For participants hospitalized in the wards, additional support questions included: How was your relationship with people during the hospitalization period? And afterward? For those in the ICU: How did you feel when you were transferred to the ICU? What does intubation mean to you? What is your perception of the ICU? Did this perception change after your hospitalization?

The interviews lasted an average of 40 minutes. The statements were recorded using an electronic device, transcribed in full, and returned to the participants for verification of any possible information omissions via WhatsApp®. It is important to note that no participant requested the exclusion of information provided during the interviews.

### Data Treatment and Analysis

The collected data were coded using the software MAXQDA Plus 2022 Student version 22.0.1, which facilitated the transcription of audio recordings, coding, and the construction of memos and diagrams necessary for the development of GT.

Following the open coding process, the data were analyzed line by line, and preliminary codes were assigned to them to understand the meanings and experiences of the research participants. Some codes had common meanings, which allowed them to be regrouped into concepts, establishing a word that represented the action recorded in the data. In total, 1,421 initial codes emerged from the analysis process.

Subsequently, focused coding began, involving the classification, integration, synthesis, and organization of codes into categories and subcategories. This process resulted in 33 focused codes, eight subcategories, and two categories, all formed to capture the key phenomenon or category of the study. All themes were derived from the data after the analytical process.

The theoretical model, constructed from the data analysis, was validated by three participants selected through a random draw conducted in Microsoft Excel, with one participant from each sampling group. They were contacted and agreed to participate in the validation, which occurred via video communication using Google Meet. During this process, the diagram of the phenomenon and its categories, the study’s objective, and the main findings of each category were presented. The validators were asked to describe their understanding of the diagram and to evaluate whether their experiences were represented. All participants felt satisfied and identified with the theory.

## RESULTS

The first and second sampling groups consisted of patients hospitalized in general wards, totaling 13 participants (eight women and five men) aged between 24 and 66 years. Seven of them had a high school education, and the others had a higher education. Most participants worked autonomously. The hospitalization period in the general ward ranged from four to 30 days.

The third sampling group included six participants who were hospitalized in the ICU, comprising four women and two men, aged between 33 and 55 years. Most of them had a high school education. The average hospitalization period was between 10 and 57 days.

**Figure 1:**
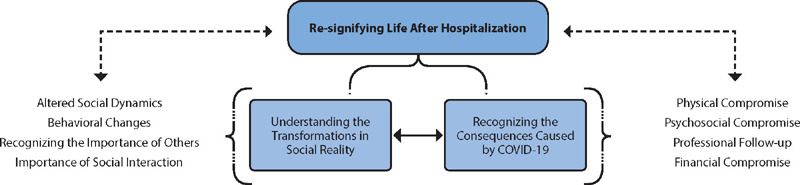
Representative Diagram of the Category “Re-signifying Life After Hospitalization”, from Research Data Using MAXQDA Software, Brazil, 2023

The study phenomenon, along with its two categories and subcategories, is presented in Figure 1.

### Understanding Transformations in Social Reality

The study participants’ understanding of the meaning of life after hospitalization for COVID-19 was that the disease would become a part of their lives and daily routines. Their interpretation of the future of the disease, which oscillated between a tendency to worsen and improve, can be explained by SI. This perspective is related not only to the experience of hospitalization but to all of the participants’ life experiences. The severity of COVID-19 and ICU hospitalization were not sufficient to impart a pessimistic outlook on the future.

[...] *we always have to be careful. If this one came, another one will come. It has always been like this; the tendency is to get worse, not better.* (WARD 13)

[...] *I believe that gradually we will adapt to everything that is happening* [...] *the way of living alongside the disease until everything calms down. It will always be there, but we will manage to mitigate and live with the disease.* (ICU 15)

The behavioral changes were considered necessary by the participants, such as not leaving home without a mask, going out less, avoiding visiting friends, not attending parties, following rules, and becoming aware of the disease and its consequences. This perspective indicates the need for participants to adopt resilient behavior in the face of the challenges posed by the pandemic.

*I don’t leave without this mask here for anything. I hardly leave the house. It is very difficult for me to go to the market to shop; it is very difficult for me to go to a park or restaurant. In fact, since I got sick, I haven’t been to a restaurant anymore. I restricted my outings a lot* [...]. (WARD 13)

[...] *I have friends that I don’t even know if they are alive. I don’t visit anyone anymore. Until this passes, I will not visit, I will not attend parties, I will not do anything. It has changed a lot, and I can’t leave the house without the mask. Sometimes I even wear it at home.* (WARD 8)

Additionally, participants realized the value of various situations that were previously considered common and began to appreciate them more, changing their priorities and reflecting on the value of health and the desire to resume an active life after recovery.

[...] *there are some things that matter more, things we need to value more, the more serious things that are worth it. Things you were afraid of before—I was afraid of flying—now I intend to fly. I intend to do different things.* (ICU 17)

[...] *I hope my health returns 100%, that I can, for example, go to the gym without fear, take a dance class without fear, go to the cinema, go to the theater, go to a bar with my friends without this fear I have today of getting re-infected.* (ICU 18)

Thus, it is evident that hospitalization led the participants to reconsider the meaning of life and the people around them. This is expected from the perspective of SI, as experiencing a particular situation results in new interpretations of that situation and other interactions within the process.

*What’s the point of arguing over such trivial things? I used to create conflicts over such insignificant matters when there are lives that matter so much more than that.* (WARD 11)

[...] *My family is a priority; it is fundamental. So, I think I started to put things in the right order. People are more important than anything else, you know. Maybe I didn’t see it that way before.* (WARD 2)

The understanding of the importance of others in their lives, society, and different daily events was reinterpreted based on their experiences with COVID-19 and the environment they were in.

### Recognizing the consequences caused by COVID-19

Numerous complications were reported due to COVID-19 and hospitalization. Some participants mentioned physical complications, such as body aches, hair loss, skin lesions, and respiratory problems, which affected their perception of the disease and the hospitalization period. This highlights the importance of reassessing the situation after discharge, as complications persist.

[...] *my hair started falling out. I was in despair over how much hair was falling out. At first, I started crying because I didn’t know whether to wash my hair or not. The floor at home was covered in hair, and I thought I was going to go bald.* (WARD 7)

[...] *I need to be strong to get through this moment and do everything for myself* [...] *my conflict is with myself, putting up the impossibilities of recovering due to having gone through this moment.* (ICU 16)

Participants emphasized the importance of professional follow-up to help them recover and return to normal life, especially for those who were in the ICU. Medical treatment and recovery from COVID-19 can influence how people see themselves and approach the process of overcoming the illness.

[...] *I had home treatment with a nurse, physiotherapist, and speech therapist, all at home, from September to December* [...] *that was when the bedsores healed, and I gradually recovered. That’s when I realized I was truly victorious, having overcome things I never imagined.* (ICU 17)

*COVID-19 and the treatment taught me patience. I had hyperbaric chamber sessions for the bedsores. I spent 90 afternoons over five months in the chamber, lying down for an hour and a half. So, maybe I developed more patience because it was a slow transition until I could say I am a normal person.* (ICU 19)

However, many participants reported a lack of guidance, professional follow-up, and access to health services after discharge, which made recovery slower and more painful. This underscores the importance of healthcare systems and social support, and how the lack of access to these resources can be distressing.

[...] *I had to seek physical therapy, I had changes in my diabetes because I take insulin* [...] *I was left without any assistance post-COVID, and it’s scary, like someone throwing you out of the house and saying, ‘now you’re on your own.* (ICU 15)

[...] *I had no assistance whatsoever. I didn’t know what to do. I didn’t have anyone with me to guide or show me what to do.* (WARD 5)

Additionally, psychological consequences such as depression, anxiety, insecurity, and shame were also mentioned after discharge, affecting interpersonal relationships. These factors suggest changes in how these individuals see themselves and communicate, as communication is a crucial social element for reestablishing meanings in the social context of SI.

[...] *I no longer have the confidence to talk with authority about what I’m saying. I’m afraid, I’m embarrassed, so I talk less. I don’t like to go out because of the way I walk or the things I say. To get things done, I need my daughter’s help; I have to write everything down and set reminders on my phone. Everything in my life has changed* [...]. (ICU 15)

[...] *it has never been the same. I feel anxiety; whenever I feel anxious, these pains come. Overall, it has changed a lot because I have insecurities about many things, and my mental state is shaken* [...]. (WARD 12)

Financial consequences were also highlighted, such as job loss or difficulty returning to work due to the disease’s sequelae, as well as expenses for medications needed for recovery. However, this experience was noted as being temporary and surmountable, as hospitalization contributed to the reinterpretation of life and priorities.

[...] *I left the hospital with a shaken mental state. When I returned, I lost my job. I have four children, so my mental state was shaken. But I knew it would pass and that it was nothing compared to what I went through during hospitalization* [...]. (WARD 12)

[...] *financially, we spent a lot of money, spent what we didn’t have, but at the time, it was a priority.* (WARD 5)

Thus, the data illustrate how people attributed new meanings to different aspects of their lives, such as physical health, mental health, finances, and identity, and how these affect their actions and interactions in a new post-pandemic social reality. SI helps us understand how individual experiences are shaped by the social and cultural context in which they occur, perceiving the construction of a new social reality.

## DISCUSSION

Based on the results evidenced in this research, the meanings attributed to certain symbols after hospital discharge were expressed, representing a process of change based on interactions with others as well as with oneself. Experiencing this process symbolized a new beginning for the participants in this study, directly impacting their daily, work, and social activities.

In Japan, for example, the pandemic motivated study participants to change their lifestyles, pay more attention to their lives and the people around them, and take preventive measures against infection, such as social and physical distancing^(15)^. In a phenomenological study conducted in Australia, it was reported that this experience led to the adoption of new behaviors, including health practices, to improve their lifestyle and health^(16)^.

Feelings of gratitude and positive emotions in people who have gone through an extreme adverse life event are known as Post-Traumatic Growth^(17)^. This growth can be associated with the prioritization of intrinsic values, such as spending more time with family or engaging in activities that bring joy. Therefore, as intrinsic values are reprioritized, concerns related to achievements become less valued^(18)^.

Another fundamental point is social support as a protective factor for the health and well-being of individuals after COVID-19^(19)^. However, in its absence, COVID-19 survivors may develop social anxiety, stress, and stigma, thus diminishing their coping resources^(20)^. Therefore, family, friends, and health professionals play an important role in these people’s support network^(21)^.

Regarding stigma, a study conducted in Iran with patients after COVID-19 discharge stated that even after fully recovering, others still avoided them. This behavior leads to a more challenging experience than the physical pain of the disease. This phenomenon can be called COVID-19 stigma^(22)^.

Corroborating our results, it has been shown that post-discharge follow-up by a multidisciplinary team is of great importance^(23)^. There are several studies on the home follow-up of patients after hospital discharge by physiotherapists^(24)^, nurses^(25)^, nutritionists^(26)^, and other professionals, all of whom seek to restore people’s health after COVID-19.

A study conducted in New Jersey highlights the importance of case management, with the development of an individual care plan after COVID-19, due to the various symptoms these patients may present and their daily variations amid the uncertainties about the disease^(27)^. This aligns with other studies that clearly indicate the need for regular monitoring of patients after discharge to assess clinical status, as well as the need for care and/or rehabilitation^(28-29)^.

However, there are challenges faced by the Unified Health System (SUS) and the Health Care Network (RAS) in caring for people with complications arising from COVID-19. These challenges are related to the available physical and human resources, inadequate structures, network disorganization and service flow, lack of coordination, and financial resources, weakening the care provided^(30)^.

In this context, the importance of territorial care and the matrix support of primary health care teams stands out. Matrix support is one of the technologies aimed at ensuring effective care within the Health Care Network (RAS), offering support to territorial care and enabling health care teams to tackle practical challenges, such as care fragmentation, training focused on the biomedical model, changes in work relationships, and different practices involved in health care^(31)^.

Among the various professionals involved, the importance of nursing care for people who have been hospitalized for COVID-19 is highlighted, as it provides a comprehensive understanding of the person in their entirety—whether psychological, social, spiritual, or biological—with quality and integrality^(32)^.

Another aspect observed in participants’ reports was the financial impact. In the United States, a study highlighted that 35% of people did not return to work within 14 to 21 days after discharge^(33)^. In Australia, in 2020, 36% of respondents experienced at least one work market shock due to COVID-19, with 34% reporting insufficient funds to meet their financial needs and 29% unable to cope with a major unexpected expense^(34)^. Therefore, unemployment and financial stress experienced by survivors post-discharge are significant social issues.

Our results demonstrated that the meanings attributed to the hospitalization experience and the COVID-19 recovery process can impact various areas of life for those who experienced this moment. This information is significant for guiding long-term treatment and organizing health care for the population^(35)^.

Considering existing care theories and models, we highlight the Person-Centered Clinical Method, where experiences must be considered by professionals when assisting individuals, taking an integral and holistic approach. This method differs from the biomedical model, which aims only to combat the disease and not the inherent interrelations of health, illness, and the experience of illness^(36)^.

Finally, it is concluded that it was possible to understand the meanings of the changes experienced by individuals after hospitalization through the use of verbal symbols, conveying the desired message, and with these symbols, the elaboration of meanings, understanding the context of life after hospital discharge.

In this study, the meanings attributed by participants after discharge from moderate and severe COVID-19 contributed to seeing their lives differently, achieving results related to changes in social reality and recognition of the consequences caused by COVID-19. Thus, the phenomenon “Re-signifying life after hospital discharge” emerged, demonstrating that GT supported by SI was adequate for the research.

### Study limitations

This research has some limitations to consider. First, the lack of long-term follow-up with the participants makes it difficult to continuously observe changes in their lives and the evolution of the meanings attributed to their experiences. Second, there is the possibility of changes in the reality mentioned during the research, which restricts the capture of dynamics and developments after data collection that could influence the results and interpretations.

It is crucial to emphasize that despite these limitations, the study achieved its purpose by understanding the meanings attributed to the changes experienced by people hospitalized due to COVID-19. For future research that may include longitudinal follow-up, the findings provide a valuable foundation that allows for a more comprehensive and detailed view of post-discharge experiences.

### Contributions to Nursing, Health, or Public Policy

The advances and contributions resulting from understanding the meanings expressed in this study will have a significant impact on nursing practice, providing evidence of the personal and social transformations experienced after hospital discharge. These findings can help nurses approach patients holistically and empathetically, taking into account the psychological, social, spiritual, and biological aspects of care.

Furthermore, the results of this research can guide health professionals and managers, emphasizing the relevance of the meanings attributed to post-hospitalization experiences. This could influence the restructuring of health services in Brazil, encouraging the adoption of public policies that value the continuous and comprehensive monitoring of patients and improve the quality of care and support provided during recovery.

## FINAL CONSIDERATIONS

The meanings and experiences attributed to the changes that occur after hospital discharge are intrinsically linked to the appreciation of interpersonal relationships, the perception of societal impact, and the consequences that the pandemic has brought to people’s lives, beyond the physical and psychological clinical changes. Additionally, the importance of follow-up by health services after discharge is emphasized. For health professionals, it is crucial to recognize the importance of adopting a holistic perspective when addressing individuals who have been hospitalized for COVID-19, considering the uniqueness of patients during the recovery process. This comprehensive approach is fundamental to ensuring continuity of care and the well-being of patients after hospital discharge.

## Data Availability

https://doi.org/10.48331/scielodata.HBSH6I
