# Supplementary material for: Meanings attributed to changes experienced by individuals after COVID-19 hospitalization
Source: Rev Bras Enferm. 2024 Aug 30;77(Suppl 1):e20230122. doi: 10.1590/0034-7167-2023-0122 (PMC11368381; doi:10.1590/0034-7167-2023-0122)
Supplement: Translation

# Translation: Significados atribuídos às mudanças vividas por indivíduos após hospitalização pela COVID-19

**DOI:** 10.1590/0034-7167-2023-0122pt

**Authors:** Camila Harmuch, Jéssica dos Santos Pini, Paula Antunes Bezerra Nacamura, Anny Caroline Ribeiro Devechi, Vinícius Brito de Souza, Juliana Graciela Vestena Zillmer, Maria Aparecida Salci, Marcelle Paiano

**Affiliations:** I Universidade Estadual de Maringá. Maringá, Paraná, Brasil; II Universidade Federal de Pelotas. Pelotas, Rio Grande do Sul, Brasil

**Keywords:** COVID-19, Acontecimentos que Mudam a Vida, Hospitalização, Teoria Fundamentada, Enfermagem

## Abstract

**Objetivos:**

compreender os significados atribuídos às experiências vivenciadas por indivíduos após hospitalização pela COVID-19.

**Métodos:**

estudo qualitativo, com referencial teórico no Interacionismo Simbólico e metodológico na Teoria Fundamentada nos Dados. Foram entrevistados 19 participantes com a forma moderada e grave da COVID-19 após hospitalização. A coleta dos dados aconteceu entre abril e novembro de 2021, mediante entrevista online, e os dados foram analisados seguindo a codificação inicial e focalizada, no software MAXQDA.

**Resultados:**

os dados ilustram novos significados a diferentes aspectos da vida após hospitalização, sendo na relação com o outro, com o meio, com a saúde física e mental, finanças, identidade e interações com uma nova realidade social.

**Considerações Finais:**

os significados estão intrinsecamente ligados à valorização das relações interpessoais, à percepção de seu impacto e às consequências após hospitalização, permitindo aos profissionais perceber a importância dessas informações para melhorar o cuidado e se preparar para enfrentar futuras epidemias.

## INTRODUÇÃO

As experiências dos indivíduos durante a hospitalização pela COVID-19 foram impactadas por vários fatores, como a presença de sintomas, a duração e o estágio da internação, a gravidade da doença, as mudanças sociais, o acesso a informações e as experiências anteriores^(1-2)^.

Nelas, foram prevalentes sentimentos como medo, negação, estigma e sensação de abandono, principalmente no início dos sintomas, na admissão hospitalar e no isolamento, juntamente com incerteza, preocupação e expectativas em relação ao prognóstico após a confirmação do diagnóstico. A aceitação da situação clínica e do tratamento ocorreu ao longo da internação e próximo à alta hospitalar^(2)^.

Durante a hospitalização, as reações do corpo também foram influenciadas pela experiência vivida, refletindo-se em uma atenção excessiva aos sintomas, mudanças na dieta, no sono e no comportamento. Em alguns casos, a doença resultou em crescimento psicológico e mudanças na abordagem dos desafios diários, devido à valorização da vida, da família, da bravura e da tenacidade^(3)^.

Em relação à gravidade da COVID-19, que está associada aos sintomas clínicos, um estudo qualitativo com 24 pacientes com COVID-19 revelou que, durante a hospitalização, aqueles com doença leve a moderada enfrentaram incertezas em relação à evolução, ao manejo clínico e à recuperação, enquanto os casos mais graves lidaram com essas mesmas questões acrescidas do medo da morte^(2)^. Ainda, os participantes internados na Unidade de Terapia Intensiva (UTI) expressaram a necessidade de compreender o que ocorreu durante esse período, uma vez que o medo, a angústia e as dores persistiram por meses após a alta^(4)^.

As pessoas que passaram por uma hospitalização devido à COVID-19 tornaram-se extremamente vulneráveis, com novas necessidades de saúde após esse período^(5-6)^, tais como síndrome de estresse pós-traumático, depressão e redução da qualidade de vida^(7-8)^. Essas novas demandas tornam a assistência a essa doença mais desafiadora, tanto para o paciente e seus familiares, quanto para os serviços de saúde^(9)^.

Para isso, considera-se que as experiências são interpretadas de acordo com os símbolos que surgem em uma situação específica, única para cada pessoa e suas interações sociais. Portanto, a teoria do Interacionismo Simbólico (IS) permite explicar os processos dinâmicos que ocorrem nas relações entre o indivíduo e os elementos de interação após a hospitalização^(10)^.

Para coletar e analisar as informações, com o objetivo de compreender profundamente os significados e as experiências pessoais complexas que surgem com a hospitalização por COVID-19, a Teoria Fundamentada nos Dados (TFD) é utilizada. Através dela, é possível obter insights aplicáveis aos cuidados de saúde, intervenções e políticas, tornando-os mais eficazes e centrados no paciente. Assim, o método permite uma abordagem holística para a pesquisa, sendo vital para compreender completamente o impacto da hospitalização pela COVID-Ί9 na vida das pessoas e como a equipe de saúde pode apoiá-las da melhor forma possível.

Nessa perspectiva, ao identificar as experiências vivenciadas pelas pessoas hospitalizadas pela COVID-Ί9, bem como seus significados, é possível estabelecer uma gestão mais eficaz dessa enfermidade e qualificar a assistência à saúde, durante e após a hospitalização. Isso, por sua vez, contribui para facilitar um retorno eficaz e oportuno dessas pessoas às suas rotinas de vida pré-pandêmicas.

## OBJETIVOS

Compreender os significados atribuídos as experiências vivenciadas por indivíduos após hospitalização pela COVID-19.

## MÉTODOS

### Aspectos éticos

O desenvolvimento do estudo ocorreu em conformidade com os preceitos éticos dispostos na Resolução 466/12, do Conselho Nacional de Saúde. Foi aprovado pelo Comitê Permanente de Ética em Pesquisa com Seres Humanos, como parte de uma pesquisa de Coorte. Todas as entrevistas foram consentidas e assinado o Termo de Consentimento Livre e Esclarecido.

Os entrevistados foram identificados pelo setor de internamento, seguido por algarismos arábicos, conforme a ordem de realização das entrevistas (“enfermaria 1”, “enfermaria 2”, “UTI 1”, “UTI 2”), garantindo o sigilo e anonimato durante todo o processo de pesquisa. A opção pelo setor de internação permite identificar nos depoimentos a gravidade do quadro do participante, visto que os pacientes mais graves foram internados na UTI.

### Referencial teórico-metodológico

Utilizou-se a IS como perspectiva teórica para dar suporte ao desenvolvimento da pesquisa e ajudar na compreensão dos diferentes processos interativos sociais e intrapessoais dos indivíduos hospitalizados pela COVID-Ί9. Por conseguinte, o uso da metodologia da TFD^(11)^ justifica-se por possibilitar uma compreensão aprofundada de áreas pouco exploradas e permitir uma análise minuciosa em relação ao fenômeno em estudo.

### Tipo de estudo

Estudo de abordagem qualitativa, realizado a partir da diretriz proposta pelo *Consolidated Criteria for Reporting Qualitative Research* (COREQ)^(12)^.

### Cenário do estudo

O estudo foi realizado em um município de médio porte localizado na região Noroeste do Paraná, com uma população de 409.657 pessoas e uma área territorial de 487,012 km^2^, de acordo com o Censo de 2022^(13)^. O número de casos confirmados de COVID-19 na região, até 31 de dezembro de 2020, era de 34.797 casos, com 26.502 recuperados e 537 óbitos. O número de internações de adultos e idosos, em 2020, foi de 1.830^(14)^.

### Participantes e Fonte de dados

Os participantes foram adultos residentes no município de estudo, que receberam alta hospitalar após serem internados em UTI ou enfermarias. A amostra foi selecionada seguindo o conceito de amostragem teórica proposto pela TFD. O processo de amostragem teórica ocorreu por meio da análise comparativa constante dos dados, direcionando o pesquisador à coleta de informações específicas para preencher lacunas conceituais. A amostragem teórica não é predeterminada, sendo definida durante a análise dos dados, na dependência da necessidade evidenciada pela teoria e pela saturação teórica dos dados. Ou seja, à medida que ocorre a repetição de informações e não são encontrados novos dados em determinado grupo, cessa-se a busca^(11)^. Ao final desse processo, a amostra foi composta por 19 indivíduos.

Para a formação dos grupos amostrais, os critérios foram estabelecidos com base no processo de amostragem teórica proposto pela TFD. Foram constituídos três grupos amostrais. Para a seleção do primeiro grupo amostral, foram adotados os seguintes critérios de inclusão: adultos que tinham sido hospitalizados e recebido alta de enfermarias entre os meses de março de 2020 a junho de 2020, com classificação final como COVID-19 moderada ou grave, e que possuíam número de telefones válidos. Foram excluídos aqueles que não atenderam às chamadas telefônicas após três tentativas em dias consecutivos.

O primeiro grupo amostral foi composto por sete participantes e teve como objetivo compreender suas experiências durante o processo de hospitalização. À medida que a coleta e análise dos dados avançavam, surgiram questões sobre a influência dos meses em que ocorreu a hospitalização. Isso se deve ao fato de que, ao longo do ano de 2020, houve oscilações entre estabilidade e picos da doença, o que poderia estar relacionado às experiências apresentadas.

Assim, optou-se por continuar a coleta de dados com participantes que haviam sido hospitalizados em enfermaria, mas em meses diferentes do primeiro grupo amostral, a fim de compreender com maior profundidade o fenômeno em estudo. Portanto, o segundo grupo amostral foi composto por seis indivíduos hospitalizados em enfermaria nos meses de setembro a dezembro de 2020, seguindo os mesmos critérios de inclusão do primeiro grupo amostral.

Com a continuidade das novas experiências dos entrevistados, optou-se pela formação do terceiro grupo amostral e levantou-se a seguinte hipótese: além do período de internamento, o setor de hospitalização tem potencial para influenciar os significados atribuídos ao adoecimento, internação e alta hospitalar? Assim, para o terceiro grupo amostral, estabeleceu-se o seguinte critério de inclusão: indivíduos classificados como COVID-19 moderada ou grave, com número de telefones válidos, que tenham sido hospitalizados na UTI em qualquer mês do ano de 2020 e tenham recebido alta hospitalar.

Foram contatados 46 possíveis participantes nos três grupos amostrais via telefone, sendo que 17 foram excluídos por não atenderem após três tentativas de contato, oito recusaram participar da pesquisa por falta de tempo e interesse, e dois não possuíam condições mentais e físicas para participar, segundo relato próprio ou de representante familiar.

### Coleta e organização dos dados

A lista dos possíveis participantes foi elaborada a partir do banco de dados de um estudo prospectivo de Coorte com indivíduos que foram hospitalizados devido à COVID-19. Esse banco de dados continha todas as informações da ficha de notificação compulsória da doença, e os critérios de inclusão e exclusão estabelecidos foram aplicados para sua elaboração. A pesquisadora, uma enfermeira capacitada para coleta de dados, entrou em contato com os possíveis participantes por telefone, estabelecendo vínculo e explicando as razões e interesses da pesquisa. Em seguida, apresentou os objetivos e o método de coleta de dados, convidando-os a participar.

Após o aceite, foi agendada uma entrevista, realizada por meio de chamada de vídeo do aplicativo *WhatsApp*®, conduzida de forma individual pela pesquisadora principal. Foi solicitado ao participante que estivesse em um ambiente privativo e tranquilo, de sua preferência, para facilitar a comunicação e tornar mais confortável sua participação. As reflexões e expressões dos entrevistados foram registradas em um diário de campo e o áudio das entrevistas foi gravado, após consentimento dos participantes. Ressalta-se que a realização das entrevistas de forma online, por meio de recurso tecnológico, se deu em atendimento às orientações do Comitê de Ética em Pesquisa, cuja recomendação de biossegurança era minimizar o risco de contágio por COVID-19 durante a coleta de dados.

A coleta dos dados do primeiro grupo amostral ocorreu entre abril e junho de 2021; do segundo grupo, entre julho e setembro de 2021; e do terceiro, entre outubro e novembro de 2021. Utilizou-se a seguinte questão norteadora: “O que mudou na sua vida após a alta hospitalar por COVID-19?”, e questões de apoio comuns a todos os grupos amostrais: O que significou o internamento para você? O que significou a alta hospitalar para você? Que mudanças aconteceram na sua vida após o diagnóstico e o internamento? Como enfrentou essas mudanças? Como está sendo para você após a alta? Como está a sua relação com as outras pessoas?

Para os participantes hospitalizados na enfermaria, acrescentou-se as questões de apoio: Como foi a sua relação com as pessoas durante o período de internamento? E após? E para os da UTI: Como você se sentiu no momento em que foi transferido para a UTI? O que significa a intubação para você? Qual a sua visão sobre a UTI? Houve mudança dessa visão após o internamento?

As entrevistas tiveram duração média de 40 minutos. Os depoimentos foram gravados com o auxílio de um dispositivo eletrônico, transcritos na íntegra e devolvidos aos participantes para verificação de possíveis supressões de informações, através do *WhatsApp*®. É importante ressaltar que nenhum participante solicitou a exclusão das informações fornecidas nas entrevistas.

### Tratamento e análise dos dados

Os dados coletados foram codificados usando o *software* MAXQDA Plus 2022 Student versão 22.0.1, que possibilitou a realização do processo de transcrição de áudios, codificações e construção de memorandos e diagramas necessários para o desenvolvimento da TFD.

Seguindo o processo de codificação aberta, os dados foram analisados linha por linha, e códigos preliminares foram atribuídos a eles, buscando compreendê-los por meio dos significados e experiências dos participantes da pesquisa. Alguns códigos tinham significados comuns, o que permitiu que fossem reagrupados em conceitos, estabelecendo uma palavra que representasse a ação registrada nos dados. No total, emergiram 1421 códigos iniciais do processo de análise.

Posteriormente, iniciou-se a codificação focalizada com um processo de classificação, integração, sintetização e organização dos códigos em categorias e subcategorias. Surgiram 33 códigos focalizados, oito subcategorias e duas categorias, formadas para alcançar o fenômeno ou categoria-chave do estudo. Todos os temas foram derivados dos dados após seu processo analítico.

O modelo teórico, construído a partir da análise de dados, foi validado por três participantes, selecionados em sorteio realizado no *Microsoft Excel,* sendo um participante de cada grupo amostral. Eles foram contactados e aceitaram participar da validação, que ocorreu por meio do serviço de comunicação por vídeo *Google Meet.* Nesse momento, foi apresentado o diagrama do fenômeno e suas categorias, o objetivo do estudo e os principais achados de cada categoria. Foi solicitado ao validador para descrever como entendeu o diagrama e avaliar se suas experiências foram representadas. Todos se sentiram satisfeitos e identificados com a teoria.

## RESULTADOS

O primeiro e segundo grupo amostral consistiram em pacientes hospitalizados em enfermaria, totalizando 13 participantes (oito mulheres e cinco homens), com idades entre 24 e 66 anos. Sete deles tinham Ensino Médio e os outros tinham Ensino Superior. A maioria dos participantes trabalhava de forma autônoma. O período de hospitalização na enfermaria variou de quatro a 30 dias.

O terceiro grupo amostral incluiu seis participantes que foram hospitalizados na UTI, sendo quatro mulheres e dois homens, com idades variando de 33 a 55 anos. A maioria deles tinha Ensino Médio. O tempo médio de hospitalização foi de 10 a 57 dias.

O fenômeno do estudo, juntamente com as duas categorias e suas subcategorias, está apresentado na Figura 1.

**Figura 1:** Diagrama representativo da categoria “Ressignificando a vida após hospitalização”, a partir dos dados da pesquisa no *software MAXQDA*, Brasil, 2023

### Compreendendo as transformações na realidade social

As compreensões dos participantes do estudo sobre o significado de viver após a hospitalização pela COVID-19 foram de que a doença faria parte de suas vidas e do cotidiano. A interpretação do futuro da doença, que oscilou entre a tendência a piorar e a melhorar, pode ser explicada pelo IS, relacionado não apenas à vivência da hospitalização, mas sim a todas as experiências dos participantes em suas vidas, já que a gravidade da COVID-19 e a internação em UTI não foram suficientes para imprimir um olhar pessimista ao futuro.

[...] *a gente sempre tem que estar se cuidando, se veio essa vai vir outra, sempre foi assim, a tendencia é piorar, não é melhorar.* (ENFERMARIA 13)

[...] *eu acredito que gradativamente nós vamos nos adaptando a tudo que está acontecendo* [...] *a maneira de viver paralelamente com a doença até que esteja tudo mais calmo, ela vai estar sempre ali, mas vamos conseguir amenizar e conviver com a doença.* (UTI 15)

As mudanças de comportamento foram consideradas necessárias pelos participantes, como não sair de casa sem máscara, sair menos, evitar visitar amigos, não frequentar festas, seguir as normas e conscientizar-se sobre a doença e suas consequências. Essa perspectiva indica a necessidade de os participantes terem um comportamento resiliente diante dos desafios enfrentados na pandemia.

*A máscara, eu não saio sem isso aqui para nada, não saio de casa quase, é muito difícil eu ir ao mercado fazer compra, é muito difícil eu ir em parque, restaurante, aliás depois que eu fiquei doente eu não fui mais em restaurante, eu restringi muito a minha saída de casa* [...]. (ENFERMARIA 13)

[...] *tem amigo meu que eu nem sei se está vivo, não visito mais ninguém, enquanto não passar isso aí eu não vou visitar, não vou participar de festa, não vou em nada, foi uma coisa que mudou muito e eu não consigo sair de casa sem a máscara, até em casa as vezes eu a uso.* (ENFERMARIA 8)

Além disso, os participantes perceberam o valor de várias situações que antes eram consideradas comuns e passaram a ser mais apreciadas, alterando suas prioridades e refletindo sobre o valor da saúde e o desejo de retomar uma vida ativa após a recuperação.

[...] *tem algumas coisas que valem mais, coisas que a gente tem que dar valor as coisas mais sérias, que valem mais a pena, coisas que você tinha medo antes, eu tinha medo de andar de avião, agora eu pretendo andar, pretendo fazer coisas diferentes.* (UTI 17)

[...] *eu espero que a minha saúde volte 100%, que eu consiga por exemplo, fazer uma academia sem ter medo, ir fazer uma aula de dança sem ter medo, que volte poder ir ao cinema, poder ir ao teatro, ir ao barzinho com as minhas amigas sem ter esse medo que eu tenho hoje de me re-contaminar.* (UTI 18)

Assim, percebe-se que a hospitalização levou os participantes a repensarem o sentido da vida e das pessoas ao seu redor. Isso é esperado a partir do IS, já que experimentar uma determinada situação resulta em novas interpretações sobre ela e sobre outras interações existentes no processo.

*Do que adianta eu ficar brigando por coisas assim tão bobas, eu ficava cassando confusão por coisas assim tão insignificantes, sendo que tem vidas que valem muito mais do que isso.* (ENFERMARIA 11)

[...] *a minha família é prioridade, é fundamental, então assim, eu acho que eu comecei a colocar as coisas na ordem correta, as pessoas são mais importantes que qualquer outra coisa sabe, talvez antigamente eu não enxergasse dessa forma.* (ENFERMARIA 2)

A compreensão da importância do outro em suas vidas, da sociedade e de diferentes eventos cotidianos foi reinterpretada a partir das experiências com a COVID-Ί9 e com o ambiente em que estavam inseridos.

### Reconhecendo as consequências causadas pela COVID-19

Foram inúmeras as complicações relatadas devido à COVID-19 e à hospitalização. Alguns participantes mencionaram complicações físicas, como dores no corpo, queda de cabelo, lesões na pele e problemas respiratórios, que afetaram sua percepção da doença e do período de hospitalização. Isso ressalta a importância de reavaliar a situação após a alta, já que as complicações persistem.

[...] *começou cair cabelo entrei num desespero do tanto de cabelo que caia. Eu, no começo comecei a chorar, porque assim, eu não sabia se eu lavava o cabelo ou não, o chão em casa ficava forrado de cabelo, eu achei que ia ficar careca.* (ENFERMARIA 7)

[...] *eu preciso ser forte para atravessar esse momento e fazer tudo por mim* [...] *o meu conflito é comigo mesmo colocando as impossibilidades de me recuperar devido ter passado por este momento.* (UTI 16)

Os participantes destacaram a importância do acompanhamento profissional para ajudá-los a se recuperar e voltar à vida normal, especialmente para aqueles que estiveram na UTI. O tratamento médico e a recuperação da COVID-19 podem influenciar a forma como as pessoas se veem e encaram o processo de superação.

[...] *tive o tratamento em casa, enfermeiro, fisioterapeuta e fonoaudiólogo, tudo em casa, de setembro a dezembro* [...]*, que foi quando as escaras fecharam e fui me recuperando aos poucos. Ali eu vi que era mesmo vitoriosa, que consegui passar por coisas que eu nem imaginava.* (UTI 17)

*A COVID-19 e o tratamento tiveram que me dar paciência, eu fiz sessões na câmera hiperbárica para as escaras, eu fui 90 tardes durante 5 meses na câmara ficando deitado por uma hora e meia numa câmara. Então, talvez eu tenha desenvolvido mais a paciência porque foi uma transição demorada para hoje eu poder dizer que estou uma pessoa normal.* (UTI 19)

No entanto, muitos participantes relataram a falta de orientação, acompanhamento profissional e acesso aos serviços de saúde após a alta, o que tornou a recuperação mais lenta e dolorosa. Isso ressalta a importância dos sistemas de saúde e do apoio social, e como a falta de acesso a esses recursos pode ser angustiante.

[...] *eu tive que procurar fisioterapia, tive alteração na diabetes porque eu tomo insulina* [...] *fiquei sem assistência alguma no pós-COVID e isso é assustador, como alguém te colocar para fora de casa e diz agora você se vira.* (UTI 15)

[...] *não tinha assistência de nada, eu não sabia o que fazer, eu não tinha uma pessoa junto comigo para direcionar, para mostrar o que fazer.* (ENFERMARIA 5)

Além disso, as consequências psicológicas, como depressão, ansiedade, insegurança e vergonha, também foram mencionadas após a alta, afetando as relações interpessoais. Esses fatores sugerem mudanças na forma como essas pessoas se veem e se comunicam, já que a comunicação é um elemento social crucial para o restabelecimento de significados no contexto social do IS.

[...] *eu não tenho mais aquela postura de estar conversando e ter propriedade do que estou falando, eu tenho medo, tenho vergonha, então eu converso menos, não gosto de sair pela forma que eu estou andando ou das coisas que eu falo e para resolver as coisas preciso da ajuda da minha filha, tenho que anotar tudo e pedir ou colocar pra despertar no celular, tudo na minha vida mudou* [...]. (UTI 15)

[...] *nunca mais foi a mesma coisa, sinto ansiedade, sempre que eu sinto ansiedade vem essas dores, no mais mudou bastante porque tenho insegurança de muitas coisas e eu estou com o psicológico abalado* [...]. (ENFERMARIA 12)

Consequências financeiras também foram destacadas, como a perda do emprego ou a dificuldade de retornar ao trabalho devido as sequelas da doença, além dos gastos com medicamentos para recuperação de sua saúde. No entanto, esta experiência foi notada como sendo passageira e superável, pois a hospitalização contribuiu para a ressignificação da vida e das prioridades.

[...] *eu saí do hospital com psicológico abalado, quando eu voltei e eu perdi o emprego, eu tenho quatro filhos, aí o psicológico ficou abalado. Mas eu sabia que ia passar e que não era nada, perto do que passei na internação* [...]. (ENFERMARIA 12)

[...] *financeiramente a gente gastou muito dinheiro, gastamos o que não tinha, mas no momento era prioridade.* (ENFERMARIA 5)

Com isso, os dados ilustram como as pessoas atribuíram novos significados a diferentes aspectos de suas vidas, como saúde física, mental, finanças e identidade, e como esses afetam suas ações e interações em uma nova realidade social pós-pandêmica. O IS nos ajuda a compreender como as experiências individuais são moldadas pelo contexto social e cultural em que ocorrem, percebendo a construção de uma nova realidade social.

## DISCUSSÃO

Diante dos resultados evidenciados nesta pesquisa, os significados atribuídos a determinados símbolos após a alta hospitalar foram expressos representando um processo de mudança com base nas interações com outras pessoas, bem como consigo mesmo. Vivenciar esse processo simbolizou um recomeço para os participantes da presente pesquisa, impactando diretamente suas atividades diárias, laborais e sociais.

No Japão, por exemplo, a pandemia motivou os participantes de um estudo a mudarem seus estilos de vida, a prestarem maior atenção à sua vida ou às pessoas ao seu redor e a realizarem as medidas preventivas contra a infecção, como o distanciamento social e físico^(15)^. Em pesquisa fenomenológica realizada na Austrália, foi referido que essa experiência levou à adoção de novos comportamentos, incluindo as práticas de saúde, a fim de melhorar seu estilo de vida e a saúde^(16)^.

Sentimentos de gratidão e emoções positivas em pessoas que passaram por um evento de vida adverso extremo são conhecidos como Crescimento Pós-Traumático^(17)^. Esse crescimento pode estar associado à priorização de valores intrínsecos, como passar mais tempo com a família ou se envolver em atividades que trazem alegria. Portanto, à medida que os valores intrínsecos são repriorizados, as preocupações relacionadas às realizações tornam-se menos valorizadas^(18)^.

Outro ponto fundamental refere-se ao apoio social como fator de proteção para a saúde e bem-estar dos indivíduos após a COVID-19^(19)^. No entanto, na ausência deste, os sobreviventes da COVID-19 podem desenvolver ansiedade social, estresse e estigma, diminuindo assim seus recursos de enfrentamento^(20)^. Assim, a família, os amigos e os profissionais da saúde possuem importante papel na rede de apoio dessas pessoas^(21)^.

Sobre o estigma, um estudo realizado no Irã com pacientes após a alta da COVID-19 afirmou que, mesmo após se recuperarem completamente, as outras pessoas ainda os evitavam. Esse comportamento leva a uma experiência mais difícil do que a dor física da doença. Esse é um fenômeno que pode ser chamado de estigma da COVID-19^(22)^.

Corroborando nossos resultados, foi demonstrado que é de grande importância o acompanhamento dos indivíduos após a alta hospitalar por uma equipe multiprofissional^(23)^. Existem diversos estudos sobre o acompanhamento domiciliar de pacientes após a alta hospitalar por fisioterapeutas^(24)^, enfermeiros^(25)^, nutricionistas^(26)^ e outros profissionais, os quais buscam o restabelecimento da saúde das pessoas após a COVID-19.

Uma pesquisa realizada em Nova Jersey destaca a importância do gerenciamento dos casos, com o desenvolvimento de um plano de atendimento individual após a COVID-19, devido aos variados sintomas que estes podem apresentar e suas variações no dia a dia, em meio às incógnitas sobre a doença^(27)^. Isso se alinha com outros estudos que indicam claramente a necessidade de monitoramento regular de pacientes após a alta para avaliar o estado clínico, bem como a necessidade de cuidados e/ou reabilitação^(28-29)^.

No entanto, há desafios do Sistema Único de Saúde e da Rede de Atenção à Saúde (RAS) perante o atendimento das pessoas com complicações oriundas da COVID-19, relacionados aos recursos físicos e humanos disponíveis, estruturas inadequadas, desorganização da rede e fluxo de atendimento, falta de coordenação e recursos financeiros, debilitando o cuidado prestado^(30)^.

Nesse contexto, destaca-se a importância do cuidado no território e do matriciamento das equipes da atenção primária à saúde. O matriciamento é uma das tecnologias que buscam a eficácia do atendimento dentro da RAS, oferecendo apoio ao cuidado no território e permitindo que as equipes envolvidas na assistência à saúde enfrentem os desafios oriundos da prática, como a fragmentação do cuidado, mudanças na formação centradas no modelo biomédico, alterações nas relações de trabalho e nas diferentes práticas que envolvem o cuidado em saúde^(31)^.

Dentre os diversos profissionais envolvidos, destaca-se a importância do cuidado de enfermagem junto às pessoas que passaram por um período de hospitalização pela COVID-19, pois este proporciona a compreensão do ser em sua totalidade, seja ela psíquica, social, espiritual ou biológica, com qualidade e integralmente^(32)^.

Outro aspecto observado nos relatos dos participantes foi o impacto financeiro. Nos Estados Unidos, um estudo realizado destaca que a porcentagem de pessoas que não retornaram ao trabalho em 14 a 21 dias após a alta foi de 35%^(33)^. Na Austrália em 2020, 36% dos entrevistados sofreram pelo menos um choque no mercado de trabalho devido à COVID-19, onde 34% relataram não ter dinheiro suficiente para suprir suas necessidades financeiras e 29% não conseguiram lidar com uma grande despesa inesperada^(34)^. Portanto, o desemprego e o estresse financeiro que os sobreviventes experimentaram após a alta é uma questão social significativa.

Nossos resultados demonstraram que os significados atribuídos à experiência da hospitalização e ao processo de recuperação da COVID-19 podem ter impactos em diversas áreas da vida dos que vivenciaram esse momento. Essas informações têm valor significativo na orientação do tratamento a longo prazo, bem como na organização da assistência à saúde da população^(35)^.

Considerando as teorias e modelos de cuidados existentes, destacamos o Método Clínico Centrado na Pessoa, em que as vivências devem ser consideradas pelos profissionais ao assistirem aos indivíduos, a partir de um olhar integral e holístico. Esse método difere do modelo biomédico, que visa somente combater a doença e não as interrelações inerentes à saúde, doença e à experiência da doença^(36)^.

Por fim, conclui-se que foi possível compreender os significados das mudanças vividas pelos indivíduos após a hospitalização, por meio da utilização de símbolos verbais, transmitindo a mensagem desejada e, com esses símbolos, a elaboração de significados, compreendendo o contexto de vida após a alta hospitalar.

Na presente pesquisa, os significados atribuídos pelos participantes após a alta hospitalar pela forma moderada e grave da COVID-19 contribuíram para que suas vidas fossem vistas de maneira diferente, alcançando resultados relacionados à mudança na realidade social e no reconhecimento das consequências causadas pela COVID-19. Emergiu, dessa maneira, o fenômeno “Ressignificando a vida após a alta hospitalar”, pelo qual se verificou que a TFD apoiada no IS mostrou-se adequada para a pesquisa.

### Limitação do estudo

Esta pesquisa possui algumas limitações a serem consideradas. Em primeiro lugar, a falta de acompanhamento a longo prazo dos participantes, dificulta a observação contínua das mudanças em suas vidas e da evolução dos significados atribuídos às suas experiências. Em segundo lugar, destaca-se a possibilidade de alteração da realidade mencionada durante a pesquisa, o que restringe a captura de dinâmicas e desenvolvimentos após a coleta de dados, os quais poderiam influenciar os resultados e interpretações.

É crucial ressaltar que, apesar dessas restrições, o estudo alcançou seu propósito ao compreender os significados atribuídos às mudanças vivenciadas por pessoas hospitalizadas devido à COVID-19. Para pesquisas futuras que possam incluir um acompanhamento longitudinal, os achados fornecem uma base valiosa que permite uma visão mais abrangente e detalhada das experiências pós-alta hospitalar.

### Contribuições para a área da Enfermagem, Saúde ou Políticas Públicas

Os avanços e contribuições resultantes da compreensão dos significados expressos neste estudo, terão um impacto significativo na prática da enfermagem, fornecendo evidências sobre as transformações pessoais e sociais vivenciadas após a alta hospitalar. Essas descobertas podem ajudar os enfermeiros a abordar os pacientes de forma holística e empática, levando em consideração os aspectos psicológicos, sociais, espirituais e biológicos do cuidado.

Ademais, os resultados desta pesquisa podem instruir profissionais e gestores de saúde, enfatizando a relevância dos significados atribuídos às experiências pós-hospitalização. Isso pode influenciar na reestruturação dos serviços de saúde no Brasil, incentivando a adoção de políticas públicas que valorizem o monitoramento contínuo e abrangente dos pacientes e melhorem a qualidade do atendimento e do suporte oferecido durante a recuperação.

## CONSIDERAÇÕES FINAIS

Os significados e as experiências atribuídas às mudanças que ocorrem após a alta hospitalar estão intrinsecamente ligados à valorização das relações interpessoais, à percepção do impacto na sociedade e às consequências que a pandemia trouxe para a vida das pessoas, para além das alterações clínicas físicas e psicológicas. Além disso, enfatiza-se a relevância do acompanhamento pelos serviços de saúde após a alta. Para os profissionais de saúde, é crucial reconhecer a importância de adotar uma perspectiva holística ao abordar indivíduos que passaram por uma hospitalização pela COVID-19, considerando a singularidade dos pacientes durante o processo de recuperação. Essa abordagem integral é fundamental para garantir a continuidade do cuidado e o bem-estar dos pacientes após a alta hospitalar.

## Data Availability

https://doi.org/10.48331/scielodata.HBSH6I
